# Supplementary material for: The genome sequence of the Crescent Plume, Marasmarcha lunaedactyla (Haworth, 1811)
Source: Wellcome Open Res. 2023 Oct 6;8:431. [Version 1] doi: 10.12688/wellcomeopenres.20107.1 (PMC11445637; doi:10.12688/wellcomeopenres.20107.1)
Supplement: Peer review history

# Reviewer report: Reviewer response for version 1

**DOI:** 10.21956/wellcomeopenres.22266.r89686

**Published:** 2024-10-01

**Authors:** Guo-Fang Jiang

**Affiliations:** 1 Nanjing Normal University, Nanjing, Jiangsu, China

I think the assembly is pretty complete, because it has a busco of 99, and there are no obvious signal collisions in the heat map in Figure 5. Annotations don't seem to be the focus of this article, so only genes are predicted. Repeated annotation, ncrna, and gene function annotation were not done, and the predicted gene integrity was not known.

Are sufficient details of methods and materials provided to allow replication by others?

Yes

Is the rationale for creating the dataset(s) clearly described?

Yes

Are the datasets clearly presented in a useable and accessible format?

Partly

Are the protocols appropriate and is the work technically sound?

Yes

Reviewer Expertise:

Ecological genomics

I confirm that I have read this submission and believe that I have an appropriate level of expertise to confirm that it is of an acceptable scientific standard.

# Reviewer report: Reviewer response for version 1

**DOI:** 10.21956/wellcomeopenres.22266.r73640

**Published:** 2024-05-16

**Authors:** Monica Poelchau

**Affiliations:** 1 National Agricultural Library, Beltsville, Maryland, USA

Douglas Boyes and Clare Boyes present a data note on the genome assembly and annotation of the Crescent Plume, Marasmarcha lunaedactyla. As most articles of this type, it is a straightforward description of the organism, its genome and annotations. A female individual was sequenced. Standard methods were used to create a high quality resource, which includes sequence representing the W and Z sex chromosomes in addition to 29 autosomes and the mitochondrial genome. The data are deposited in the appropriate places. The article is well written and contains sufficient information to reproduce the work.

Are sufficient details of methods and materials provided to allow replication by others?

Yes

Is the rationale for creating the dataset(s) clearly described?

Yes

Are the datasets clearly presented in a useable and accessible format?

Yes

Are the protocols appropriate and is the work technically sound?

Yes

Reviewer Expertise:

Genomics, databases

I confirm that I have read this submission and believe that I have an appropriate level of expertise to confirm that it is of an acceptable scientific standard.
